# Therapeutic effect of an ayahuasca analogue in clinically depressed patients: a longitudinal observational study

**DOI:** 10.1007/s00213-021-06046-9

**Published:** 2022-01-24

**Authors:** Kim van Oorsouw, S. W. Toennes, J. G. Ramaekers

**Affiliations:** 1 Faculty of Psychology and Neuroscience, Department of Clinical Psychological Science, Maastricht University, Maastricht, The Netherlands; 2 Institute of Legal Medicine, Goethe University of Frankfurt, Frankfurt, Germany; 3 Faculty of Psychology and Neuroscience, Department of Neuropsychology and Psychopharmacology, Maastricht University, Maastricht, The Netherlands

**Keywords:** Anahuasca, Field study, Clinical, Beck Depression Inventory, Anxiety, Stress, Mindfulness, Ego dissolution, Oceanic boundlessness

## Abstract

**Rationale:**

Studies have suggested mental health improvements following the use of the psychotropic plant concoction ayahuasca in non-clinical and clinical samples.

**Objectives:**

The present observational study assessed depressive symptomatology in 20 clinically depressed patients (symptom score > 13 on the Beck’s Depression Inventory) before attendance of an ayahuasca ceremony and 1 month and 1 year after. Secondary measures included ratings of altered states of consciousness and ego dissolution during the ayahuasca ceremony as well as global measures of mindfulness, satisfaction with life, depression, anxiety, and stress.

**Results:**

Twenty participants completed baseline and 1-day follow-up, 19 completed measures at 1-month follow-up, and 17 completed measures at 1-year follow-up. BDI scores reduced from baseline (*M* = 22.7) to all post-ceremony measures (*M*s 11.45, 12.89, and 8.88, for 1-day, 1-month, and 1-year follow-up, respectively). After 1 day, 12/20 participants were in remission (BDI < 13). Remission rates after 1 month and 1 year were 13/19 and 12/17, respectively. Three participants remained mildly depressed (BDI 14–19) at the 1-month and 1-year follow-up. Two participants did not respond and remained at a moderate/severe level of depression at 1-year follow-up. Reductions on the secondary mental health measures and increases in mindfulness and satisfaction with life were found up to 1 year post-ceremony. Improvements in clinical depression and mental health correlated with levels of experienced ego dissolution and oceanic boundlessness during the ceremony up to 1 month after the ceremony. Engagement in additional mental health treatments or use of another psychedelic during study participation may have contributed to improved mental health ratings at 1-year follow-up.

**Conclusion:**

Ayahuasca produces long-term mental health improvements in clinically depressed patients, which highlights its therapeutic potential.

**Supplementary Information:**

The online version contains supplementary material available at 10.1007/s00213-021-06046-9.

## Introduction

Ayahuasca is a hallucinogenic plant brew from the Amazon that contains N,N-dimethyltryptamine (DMT) and monoamine oxidase inhibitors (MAO-I) (Riba et al. 2001). DMT is a short-acting psychedelic tryptamine that is also naturally present in other plants, mammals, and human beings (Barker 2018). MAO-I enables DMT, which is usually rapidly broken down in the gut, to pass the blood–brain barrier and interact with serotonergic (5-HT) receptors in the central nervous system (Cameron and Olson 2018). The indigenous ayahuasca concoction contains the bark of the *Banisteriopsis caapi* which together with the leaves of *Psychotria viridis* have been used for healing purposes by indigenous tribes for centuries (Frecska et al. 2016; Miller et al. 2019). Other plant combinations containing DMT (e.g., *Mimosa hostilis*) and MAO-I (e.g., Peganum harmala) are regularly used in Western countries. These are called ayahuasca analogues or anahuasca. These substitutes are used because they produce similar effects as ayahuasca but are cheaper and easier to access in Europe (Kaasik et al. 2021). Although the pharmacological actions of these substitutes are similar, some report that their physical and experiential effects differ from the traditional plant combination (Kaasik et al. 2021). Kaasik and colleagues investigated a large number of traditional ayahuasca and ayahuasca analogue samples and found that ayahuasca analogue samples were generally higher in DMT and harmala concentrations. Yet, also within the traditional ayahuasca brews a large variation can be found in beta carboline ratios (see Kaasik et al. 2021; Uthaug et al. 2018).

Ayahuasca or ayahuasca analogue concoctions are usually ingested in a group setting guided by a ceremonial leader. About 30–45 min after ingestion, participants start experiencing visions and altered states of consciousness, which can last between 4 and 6 h. Frequently reported adverse effects are nausea and vomiting (Dos Santos 2013), which are considered part of the “cleansing” (Shanon, 2014). Overall, the use of ayahuasca is safe and non-addictive when taken in a supervised setting (Barbosa et al. 2016; Bouso et al. 2012; Dos Santos et al. 2012; Netzband et al. 2020). Yet, some reports associate ayahuasca or some of the (anahuasca, e.g., harmala) alkaloids with higher risks of serious intoxication (Dos Santos 2013; Kaasik et al. 2021; Ott 1999). Combinations with other additives (e.g., 5-MeO-DMT; Sklerov et al. 2005) may lead to fatal intoxication. Ayahuasca and anahuasca administration should be avoided in consumers with underlying predispositions to toxic reactions (e.g., cardiovascular dysfunctions, serotonergic drug use) and should never be taken in an unsupervised setting (Dos Santos 2013).

Observational studies in healthy volunteers have demonstrated antidepressant, anxiolytic, and stress reducing properties of ayahuasca in adult (Grob et al. 1996; McKenna et al. 1998; Dos Santos et al. 2016; Uthaug et al. 2018; Van Oorsouw et al. 2021) and adolescent users (Da Silveira et al. 2005). According to the World Health Organization, more than 264 million people suffer from depression of which about one-third do not respond to treatment (Conway et al. 2017; WHO 2017). The antidepressant potential of a single doses of ayahuasca has also been demonstrated in treatment-resistant depressed patients who demonstrated a rapid relieve of symptoms lasting for up to 1–3 weeks in open-label studies (De Lima Osório et al. 2015; Sanches et al. 2016; Palhano-Fontes et al. 2019) as well as in a placebo-controlled trial (Palhano-Fontes et al. 2019). Recently, an observational study reported reductions in anxiety, depression, and substance use disorder lasting up to 6 months post-intake in a sample of first time ayahuasca users that met the clinical diagnostic criteria for these disorders at baseline (Jiménez-Garrido et al. 2020). Recently, Ruffell et al. (2021) reported reductions in BDI depression scores in a sample of 63 ayahuasca ceremony attendants and a 77% remission rate in a sub-sample of 31 clinically depressed (BDI > 13) patients up to 6 months post-ceremony.

Acute effects of ayahuasca may be attributed to pharmacological changes in brain activity, although long-term changes may also be attributed to altered states of consciousness such as ego dissolution or mystical type of experiences (Dos Santos et al. 2007; Frecska et al. 2016; Griffiths et al. 2011; Van Oorsouw et al. 2021; Roseman et al. 2018; Uthaug et al. 2018). Not only ego dissolution (Nour et al. 2016) but also oceanic boundlessness (OB; Studerus et al. 2010) refer to feelings of unity and transcendence. Higher levels of ego dissolution were negatively related to levels of depression the day after drinking ayahuasca (Uthaug et al. 2018), and higher levels of oceanic boundlessness were related to lower post-ceremony depression and somatization ratings (Van Oorsouw et al., 2021). On the other hand, a higher intensity of aversive and anxious experiences (i.e., anxious ego dissolution (AED; Dittrich et al. 2010; Studerus et al. 2010) was related to higher post-ceremony anxiety reports (Van Oorsouw et al. 2021). Both OB and AED have also been found to predict mental health outcomes with other psychedelics such as psilocybin (Roseman et al. 2018), 5-Meo-DMT (Uthaug et al. 2019), and ketamine (Aust et al. 2019).

The therapeutic effects of ayahuasca may be related to changes in mindfulness. Previous studies have shown that participants reported to be more aware and less judgmental towards themselves after ayahuasca intake (Murphy-Beiner and Soar 2020; Soler et al. 2016; Uthaug et al. 2018; Van Oorsouw et al. 2021). Insights, a more positive life stance and changed worldview are commonly reported outcomes after ingestion of ayahuasca (Bouso et al. 2012; González et al. 2020; Halpern et al. 2008) and may contribute to improvements in mental health symptoms. A recent qualitative study among ceremony facilitators suggests that integration practices predict positive ceremony outcomes (Callon et al. 2021).

The present study investigated antidepressant effects of an ayahuasca analogue in clinically depressed patients after a single ceremony (consisting of two drinking rounds in one single night). Changes in depression, anxiety, stress, somatization, mindfulness-related capacities, and satisfaction with life were assessed at baseline and approximately 1 day, 1 month, and 1 year post-intake. This study adds to the existing literature by including a clinically validated measure of depression and a long term (1-year) follow-up measure. Based on previous findings, we hypothesized that participants would show improvements in all mental health variables, mindfulness, and satisfaction with life after intake. A second hypothesis was that changes in mental health are related to levels of altered states of consciousness and especially levels of ego dissolution and oceanic boundlessness.

## Methods

### Participants and procedure

Data were collected from ayahuasca ceremony attendants (*N* = 20) in the Netherlands. We asked ceremony facilitators to refer participants to us who reported treatment of depression as the main motivation for ceremony participation during intake interviews. The principal investigator contacted these individuals via e-mail, informed them about the study, and asked them to enroll prior to the ayahuasca ceremony. Study participation was voluntary and no incentives were provided. All participants were native Dutch speakers. Participants were screened by the facilitators for their physical and mental capability to drink ayahuasca analogue and received preparatory instructions (e.g., to abstain from processed food and alcohol at least 4 days prior to participation) and any (non-prescribed) drugs. Participants who were taking anti-depressant medication had all stopped their medication at least 6 weeks before taking part in the ceremony. In case they used additional prescription medication that might interact with the ayahuasca analogue, they were instructed to stop these medications as well in close consultation with their GP or specialist. Initially, 33 participants consented to the study and completed baseline measures. The Beck Depression Inventory (BDI) score administered in the baseline online survey served as a selection criterion for study admission. Twenty participants (9 males, mean age 35.30 (*SD* = 10.10)) met the criteria for mild to severe depression (score > 13 on Beck Depression Inventory, BDI) and were included in the study. Thirteen participants did not meet the criteria (BDI scores were < 13) and were not included in the present study.

All participants took part in the study because they suffered from chronic depression (> 2 years). All had been or were still receiving psychological treatment for their depression at baseline. Eighteen out of 20 participants had received anti-depressant medication in the past but claimed that they stopped because of lack of efficacy or side effects. Nineteen participants were new to drinking the ayahuasca analogue; one had taken ayahuasca analogue on 2 previous occasions.

Study participants took part in 4 test sessions. After being informed about the study and giving their consent, participants completed an online 30-min survey in the week before they took part in an ayahuasca ceremony via a Qualtrics Survey link (baseline measure). The second survey (1 day post-ceremony) was sent to them the day after the ceremony and the third survey 1 month after the ceremony (1 month post-ceremony) and over 1 year after the ceremony (1 year post-ceremony). Participants did not always complete the surveys immediately and some had to be reminded several times. The 1-day post-ceremony ayahuasca assessment was completed within a range of 1–13 days (mean = 4.35 days (*SD* = 3.48)) and the 1 month post-ceremony assessment within a range of 31–70 days (mean = 39.00 days (*SD* = 9.30)). There was also a 12–16-month range in the 1 year post-ceremony response time (mean = 13.47 months (*SD* = 1.06)). Baseline measures were collected between November 2018 and November 2020. However, due to Covid-19 measures early 2020, our data collection was delayed. Prolonged lockdown measures resulted in cessation of data collection and the idea to include a long-term follow-up for the sample at hand. For participants that took part end 2018/early 2019 (*n* = 9), this resulted in a longer follow-up of 12–16 months. Despite the range in response time, the time points will further be referred to as “1-day follow-up,” “1-month follow-up,” and “1-year follow-up.”

This study was approved by the standing Ethical Review Committee at Maastricht University, the Netherlands. The research team was not involved in the screening, preparation, organization, administration, and supervision of the ayahuasca ceremonies that were visited.

### Ceremonial setting

The ayahuasca analogue brew was taken in a non-religious setting. All ayahuasca ceremonies were conducted in the evenings and lasted between 6 and 8 h. The ceremony leaders had a background as ayahuasquero (i.e., entheogenic plant shaman), coach, and/or (psycho)therapist. During the ceremony, two rounds of ayahuasca analogue were served to the participants. Rituals performed during the ceremony included burning herbs and/or tobacco (mapacho), playing various musical instruments or recorded music, and singing healing songs (icaros). During ceremonies, on average, 10 participants were lying down on mattresses (including buckets and paper towels). Additionally, on average, 1 caretaker per 4 participants was present to help participants if needed during the ceremony (e.g., bathroom visit). After the ceremony, participants were offered fresh fruit and/or soup. Before and after the ceremony, participants received an extensive intake, coaching, and preparation guidelines. The morning after the ceremony, experiences were shared in the group, and participants received or were referred to coaching/therapy when needed.

### Ayahuasca analogue

Participants were recruited via 3 different centers in the Netherlands that advertised with serving ayahuasca, but were in fact using ayahuasca analogue brews. All centers prepared their own ayahuasca concoction and used Peganum Harmala as a MAO inhibitor (one combined it with B-caapi) and Jurema or Mimosa containing DMT. MAO-I and DMT were given in separate drinks within a 15-min interval. At each location, the total dose was divided over 2 rounds of administration spaced about 2.5 h apart. At location 1, participants received 3 gr of *Peganum harmala* (diluted in a 50-ml drink) and 15 g of *Mimosa hostilis* (diluted in a 50-ml drink). At location 2, participants received 4 g *Peganum harmala* and 40 g *Banisteriopsis caapi* (diluted in a 60-ml brew) and 9 g Mimosa hostilis (diluted in a 60-ml drink). At location 3, participants received 5 g *Peganum harmala* (diluted in a 50 ml brew) and 16.67 g *Mimosa hostilis* (diluted in a 50-ml brew). Samples of the brews were collected in each center to determine alkaloid concentrations using high-performance liquid chromatography-mass spectrometry (LC–MS). For calibration pure reference substances of N,N-dimethyltryptamine (DMT; Cerilliant, Round Rock TX, USA) and of tetrahydroharmine (THH), harmine, and harmaline (LGC GmbH, Luckenwalde, Germany) were used.

Mean concentrations per sample as well as the estimates of the total ingested doses of the active ingredients are given in Table 1.

**Table 1:** Concentrations and estimated doses of DMT, harmaline, harmine, and tetrahydroharmine (THH) in ayahuasca analogue brews at each location. The total volume of ayahuasca analogue brew was given divided over 2 rounds of administration. n.d., not determined

Ayahuasca brew (volume, dose)	DMT (mg/ml)	Harmaline (mg/ml)	Harmine (mg/ml)	THH (mg/ml)
**Location 1 (*N* = 3)**				
*Peganum harmala* (50 ml, 3 gr)		2.44	1.32	< 0.05
*Mimosa hostilis* (50 ml, 15 gr)	1.89			
**Location 2 (*N* = 12)**				
*Banisteriopsis muricata*; *caapi* (40 ml, 40 gr)	0.62	0.30	3.28	1.72
*Peganum harmala* (20 ml, 4 gr)		1.48	0.63	< 0.05
*Mimosa hostilis* (60 ml, 9 gr)	0.83			
**Location 3 (*N* = 5)**				
*Peganum harmala* (50 ml, 10 gr)		2.43	4.46	n.d
*Mimosa hostilis* (50 ml, 16.67 gr)	1.86			
**Estimated total dose of active ingredients**	**DMT (mg)**	**Harmaline (mg)**	**Harmine (mg)**	**THH (mg)**
Location 1	94.6	122.0	66.1	1.9
Location 2	74.5	41.8	143.6	69.3
Location 3	92.9	121.4	223.0	n.d
Mean	87.4	95.1	144.2	35.6

### Measures

Baseline, 1-day, 1-month, and 1-year follow-up surveys were identical, took about 30 min to complete and consisted of a number of questionnaires. The primary outcome measure was the Beck Depression Inventory (BDI), a clinical, psychometric measure for measuring severity of depression. Secondary mental health measures included the Depression, Anxiety, and Stress Scale-21 (DASS-21; Henry and Crawford 2005), the Brief Symptom Inventory-18 (BSI-18; Derogatis 2001), the Five Facets Mindfulness Questionnaire-15 (FFMQ-15; Baer et al. 2006), and the Satisfaction with Life Scale (SWLS; Diener et al. 1985). In the 1-day post-ceremony survey, participants also completed the Ego Dissolution Inventory (EDI; Nour et al. 2016) and 5 Dimensions of Altered States of Consciousness Scale (5D-ASC; Dittrich et al. 2010; Studerus et al. 2010). Below we will only describe BDI as the main outcome measures. All other questionnaire descriptions can be found in the supplementary materials.

#### BDI

Beck’s Depression Inventory (BDI-II) is a 21-item self-report rating scale that asks about depression symptoms (Beck et al., 1996, Dutch translation Van der Does, 2002). Items are rated on a 4-point scale from 0 (symptom absent) to 3 (severe symptoms). Anxiety symptoms are not assessed but affective, cognitive, somatic, and vegetative symptoms are covered, reflecting the DSM-IV criteria for major depression. An example of the item “Loss of interest” is 0 = “I have not lost interest in other people or activities,” 1 = “I am less interested in other people or things than before,” 2 = “I have lost most of my interest in other people or things,” 3 = “It’s hard to be interested in anything.” The total score is calculated by adding the highest ratings for all 21 items. The minimum score is 0, and maximum score is 63. Higher scores indicate greater symptom severity. In non-clinical populations, scores above 20 indicate depression. In those diagnosed with depression, scores of 0–13 indicate no-minimal depression, 14–19 (mild depression), 20–28 (moderate depression), and 29–63 (severe depression) (Beck et al. 1996). In the present study, all participants with baseline scores > 13 were included. Cronbach’s alpha in the present sample was 0.91. The primary outcome measure was the change in depression severity on BDI scores from baseline to 1 day, 1 month, and 1 year post-ceremony, with scores < 13 being defined as “remission.” Furthermore, the proportion of participants meeting response rate, i.e., a reduction of 50% or more from baseline scores, was calculated.

### Follow-up interviews

After study completion, participants were contacted to inquire about their use of psychedelics and other medications, major life events, as well as cognitive or behavioral therapies during study participation. In addition, participants were asked to mention any additional events that in their opinion could have affected their mental state.

### Statistical analyses

Data was analyzed with the Statistical Package for the Social Sciences (SPSS version 25).

For the main analyses, we carried out linear mixed model analysis that included session (4 levels: baseline, immediately post, 1 month post, 1 year post-ceremony) as within subject factor. The covariance structure for the repeated measures was chosen according to best fit and could vary across outcome variables. Different covariance structures used included compound symmetry heterogeneous (CSH) as well as first lag autoregressive (AR1) structures. Significant main effects of session were followed by pairwise comparisons between baseline and post-sessions with Bonferroni adjustments for multiple comparisons. Pearson’s correlations were carried out to investigate how the level of ego dissolution (EDI), oceanic boundlessness (OB), and anxious ego dissolution (AED) during the anahuasca ceremony were related to the scores on depression, mental health related measures, mindfulness, and satisfaction with life at the post-sessions.

## Results

In total, 20 participants completed the baseline and 1-day post-ceremony survey, 19 participants completed the 1-month post-survey, and 17 participants completed the 1-year post-survey. Prior to baseline, all participants (100%) had received (psycho)therapy to treat their depression and 16 (80%) reported to have taken one or more types of antidepressant medication (e.g., venlafaxine, citalopram, paroxetine). Between the 3rd and 4th measures, 8 of them still received therapy (47%) and 3 (17%) had started taking antidepressant medication (again). Nine participants (53%) reported another experience with psychedelics (between the 1-month and 1-year measure). Of those participants, four drank ayahuasca, three took psilocybin, and two took LSD. One participant reported the use of PCP on one occasion. Overall, participants in the study expected a beneficial outcome from participating in an ayahuasca ceremony. The majority (*N* = 17) expected that their participation in the ayahuasca ceremony would reveal new insights, heal underlying pain or trauma, connect them to their “true” selves, show them the cause of their depression, and/or to make them aware of rigid thought patterns and beliefs. Only three participants had no/low expectations. They did not expect a miracle from ayahuasca since nothing had worked for them so far.

There were no apparent differences in the demographics of participants that dropped out of or completed the study.

### Mental Health

#### BDI

**Fig. 1:**
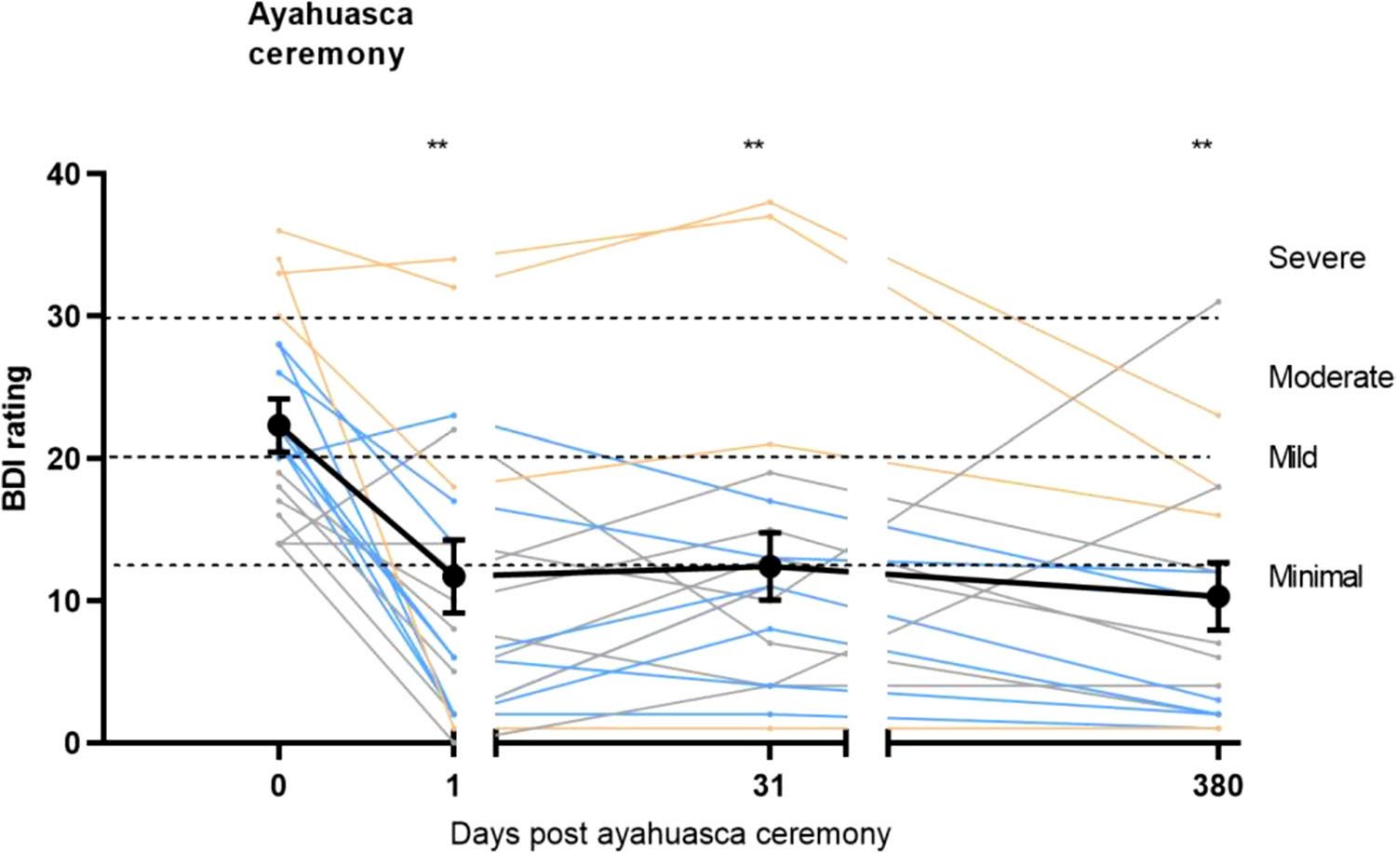
Individual and mean (SE) BDI depression scores as a function of time after ayahuasca analogue (i.e., 1-day, 1-month, 1-year). Individuals with severe, moderate, and mild depression at baseline are shown in orange, blue, and gray lines, respectively. **p < .01

Mixed model analysis for BDI depression scores revealed a significant session effect (*F*_3, 19.21_ = 11.88; *p* < 0.001). There was a significant reduction in BDI scores between baseline and all follow-up sessions (all *t*’s > 5.02, all *p*’s < 0.001, all *d*_z_ > 1.12). Figure 1 displays mean BDI scores as a function of time as well as individual BDI scores.

Table 2 shows the number and proportion of participants that scored in the BDI categories “no/minimal depression” (i.e., remission: BDI < 13), “mild depression” (BDI 14–19), “moderate depression” (BDI 20–28), and “severe depression” (BDI 29–63) at baseline, 1 day, 1 month, and 1 year post-ceremony and the response rate (> 50% reduction in depression score relative to baseline). As can be seen in Table 2, at baseline, all participants suffered from mild to severe depression (BDI > 13). One day post-ceremony, all but 2 participants (90%) showed a reduction in their depression ratings. A remission rate of 60% and a response rate of 55% were observed. One month post-ceremony, 68% of participants were in remission (response rate 47%). After 1 year, the remission rate was 71% and the response rate was 65%.

**Table 2:** Number (%) of participants within the diagnostic categories of BDI (mild, moderate, severe) and associated response and remission rates as a function of time after the ayahuasca ceremony

BDI category	Remission BDI < 13	Mild BDI 14–19	Moderate BDI 20–28	Severe BDI 29–63	Response rate ΔBDI > 50%
Baseline (*N* = 20)	0 (0%)	7 (35%)	9 (45%)	4 (20%)	0 (0%)
1 day (*N* = 20)	12 (60%)	4 (20%)	2 (10%)	2 (10%)	11 (55%)
1 month (*N* = 19)	13 (68%)	3 (16%)	1 (5%)	2 (11%)	9 (47%)
1 year (*N* = 17)	12 (71%)	3 (17%)	1 (6%)	1 (6%)	11 (65%)

There was no correlation between BDI scores and the use of psychedelics or medication between the 1 month and 1 year post-ceremony (*r* = 0.038, *p* = 0.879). Participants who used additional psychedelics did not reduce significantly more in their BDI scores between 1 month and 1 year post-ceremony than participants who did not use additional psychedelics (88% vs 62% of the participants, respectively, *Pearson χ*^2^ = 1.64, *p* = 0.20).

In a secondary analysis, baseline BDI score was included as a covariate in the model to see if baseline scores affected any of the follow-up measures and whether this differed for high/low baseline scores. To that end, centralized baseline scores were calculated where high and low BDI baseline scores were defined as centralized baseline score ± 1*SD*. The significant session × centralized BDI baseline score interaction (*F*_2, 36.45_ = 5.47; *p* = 0.008) showed that the significant session (i.e., reduction in BDI scores) effect emerged for participants with relatively high baseline scores (*F*_2, 36.55_ = 7.48; *p* = 0.002) and not for those with low baseline scores (*F*_2, 36.62_ < 1.0).

#### DASS-21

**Fig. 2:**
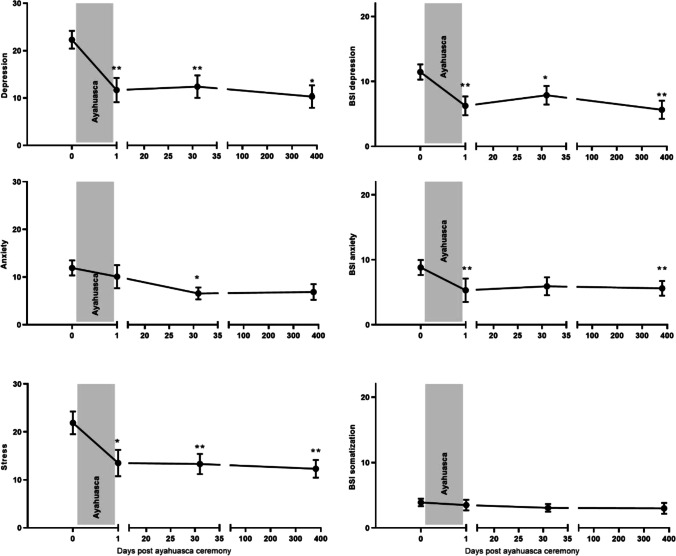
Mean (SE) subjective ratings of DASS stress, depression and anxiety, and BSI depression, anxiety, and somatization as a function of time after ayahuasca analogue (i.e., 1 day, 1 month, 1 year). *p < .05, **p < .01

For depression ratings, a significant effect of session emerged (*F*_3, 19.05_ = 8.94; *p* = 0.001). Depression scored dropped significantly between baseline and all follow-up sessions (all *t*’s > 3.78, all *p*’s < 0.008, all *d*_z_ > 0.83). For ratings of anxiety, also a main session effect was found (*F*_3, 35.28_ = 4.25; *p* = 0.012), with significant reduction between baseline and the 1-month follow-up (*t* (30.46) = 3.34, *p* = 0.015, *d*_z_ = 0.75). For stress, the significant session effect (*F*_3, 39.87_ = 6.03; *p* = 0.002) showed a significant reduction in self-reported stress between baseline and all follow-up sessions (all *t*’s > 2.94, all *p*’s < 0.035, all *d*_z_ > 0.65) (see Fig. 2).

#### BSI-18

For ratings of BSI depression, a significant session effect was found (*F*_3, 42.48_ = 11.47; *p* < 0.001) with significant reductions reported between baseline and all follow-up sessions (all *t*’s between 3.15 and 5.17, all *p*’s < 0.019, all *d*_z_ > 0.74). For anxiety ratings, also a significant session effect emerged (*F*_3, 19.70_ = 5.86; *p* = 0.005), with a significant reduction in anxiety between baseline and the 1 day post-test (*t* (20.00) = 3.80, *p* = 0.007, *d*_z_ = 1.01) and between baseline and the 1-year follow-up (*t* (20.23) = 3.71, *p* = 0.009, *d*_z_ = 0.85). No session effect was found for somatization ratings (*F*_3, 27.85_ = 1.03; *p* > 0.05) (see Fig. 2).

#### SWLS

**Fig. 3:**
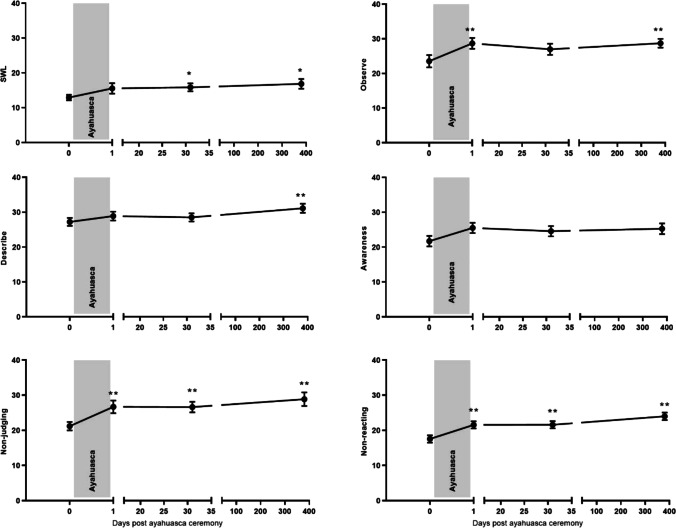
Mean (SE) subjective ratings of Satisfaction with Life (SWL), and FFMQ scales: observing, describing, awareness, non-judging, and non-reacting. *p < .05, **p < .01

Self-reported satisfaction with life did increased over sessions (*F*_3, 19.05_ = 3.57; *p* = 0.033), with a significant increase from baseline to 1-month and 1-year follow-up (all *t*’s > 3.06, all *p*’s < 0.038, all *d*_z_ > 0.71) (see Fig. 3).

#### FFMQ-15

For mindful *observing*, a main effect of session was found (*F*_3, 45.00_ = 6.62; *p* = 0.001). Self-reported mindful observing increased from baseline to the day after the ceremony, and from baseline to the 1-year follow-up (both *t*’s > 3.98, both *p*’s = 0.002, both *d*_z_ > 0.89). In addition, the significant session effect for mindful *describing* (*F*_3, 34.04_ = 4.02; *p* = 0.015) showed an increase only at the 1-year follow-up measure as compared to baseline (*t* (36.09) =  − 3.45, *p* = 0.009, *d*_z_ = 0.83). Also, significant session effects emerged for mindful *non-judging* (*F*_3, 30.16_ = 10.57; *p* < 0.001) and *non-reacting* (*F*_3, 50.57_ = 10.21; *p* < 0.001). There was a significant increase in non-judging and non-reacting between baseline and all follow-up session (all *t*’s > 3.40, all *p*’s < 0.007, all *d*_z_ > 0.77). No significant session effect emerged for mindful *awareness* (*F*_3, 53.05_ = 2.31; *p* = 0.087) (see Fig. 3).

### The psychedelic experience

#### EDI

Overall, mean (*SD*) ego dissolution rating was 44.09 (32.48). Overall, total EDI ratings varied between 0 (no dissolution) and 100 (maximal dissolution).

#### ASC

**Fig. 4:**
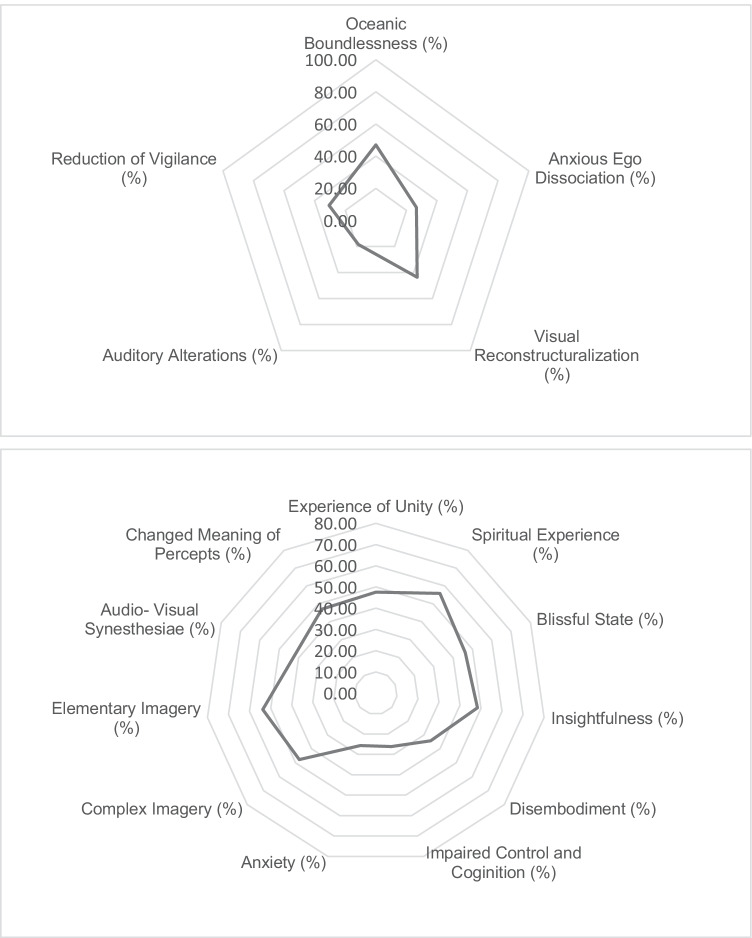
Mean ratings of the experience of altered states of consciousness as assessed with 5D-ASC (from 0 to 100%) dimensions (top) and subscales (bottom)

Mean ratings on 5D-ASC dimensions (oceanic boundlessness (OB), anxious ego dissolution (AED), visionary restructuralization (VR), auditory alterations (AA), reduction of vigilance (RV)) overall varied between 16.75 and 45.55, with mean scores on OB and AED being 45.55 (*SD* = 30.80) and 25.30 (*SD* = 22.54), respectively. For 5D-ASC subscales, means varied between 23.65 and 54.30, indicating that all participants experienced moderate levels of altered states of consciousness. All means can be found in Fig. 4. Our focus was mainly on OB and AED as a reflection of mystical vs adverse experiences.

### Relationship between the psychedelic experience and mental health changes

**Fig. 5:**
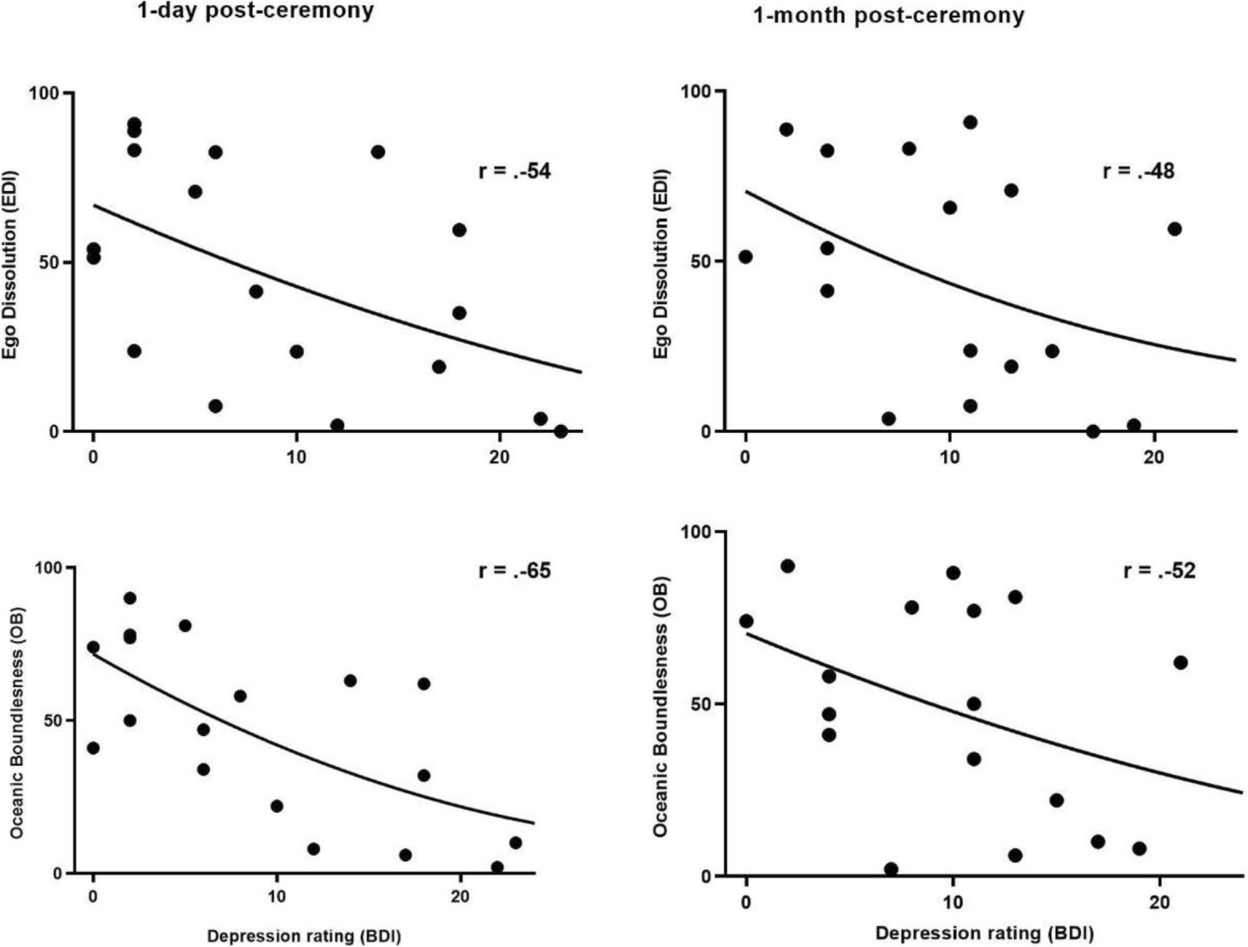
Correlations between levels of ego dissolution (EDI), oceanic boundlessness (OB), and ratings on BDI 1-day and 1 month post-ceremony

Pearson correlations were calculated between the level of ego dissolution (EDI), oceanic boundlessness (OB), and anxious ego dissolution (AED) and the primary outcome measure (BDI) 1 day, 1 month, and 1 year post-ceremony (see Fig. 5). One day after the ceremony, a significant negative correlation emerged between EDI and BDI scores (*r* =  − 0.545, *p* = 0.010). The negative correlation between EDI and BDI scores still existed at the 1-month follow-up (*r* =  − 0.455, *p* = 0.050), but was no longer related to BDI ratings at the 1-year follow-up. This indicates that higher levels of experienced ego dissolution were related to lower depression scores on BDI lasting until 1 month post-ceremony. Furthermore, 1 day post-ceremony, a significant negative correlation was found between OB and BDI depression (*r* =  − 0.650, *p* = 0.002). This negative correlation with OB persisted at the 1-month follow-up (*r* =  − 0.521, *p* = 0.022 and *r* =  − 0.482, *p* = 0.037, respectively) but not at the 1-year follow-up. These findings suggest that higher levels of OB were related to lower depression levels lasting up to 1 month post-ceremony. There were no significant correlations between BDI and AED scores at any of the follow-up measures.

### Follow-up interviews

In the year between the 1-month and 1-year follow-ups, the percentage of remissions further increased from 68 to 71%. To learn which factors had affected their depression in the year before the last measure, we conducted follow-up interviews. In total, we talked to 16 participants. Nine participants reported at the 1-year follow-up survey that they had used additional psychedelics, and three participants were taking antidepressant (AD) medication. We talked to seven out of those nine participants. As indicated above, the BDI scores did not significantly differ between participants who took additional psychedelics as compared to those who did not. Subjectively, however, five participants (71%) judged that the additional experience with a psychedelic was beneficial to their mental state.

All three participants that used additional antidepressant medication reported their use had been beneficial. Eight participants reported that they had received mental health therapy at the 1-year follow-up survey. We were able to contact seven of them after study completion. All of them reported beneficial effects of therapy on their mental health.

When we asked them what other factors may have affected their mental health, most participants (88%) reported that they made some changes in their lifestyle by the time of the 1-year follow-up of the study. Meditation, breathwork, and taking regular walks in nature were the most prevalent measures of self-directed mental health support. Some stopped using alcohol or drugs. Others went back to school or switched jobs. Dynamics in interpersonal relationships were also frequently reported as relevant moderating factor of mental health status.

## Discussion

The goal of the present study was to evaluate the effect of a single ayahuasca ceremony in 20 clinically depressed participants on levels of depression, anxiety, stress, somatization, mindfulness, and satisfaction with life 1 day, 1 month, and 1 year post-ceremony. All participants had received treatment (i.e., (psycho-)therapy and/or pharmacological treatment) in the past. At our baseline measure, all participants showed mild to severe symptoms of depression. A total of 65% showed moderate to severe symptoms of depression as assessed with the BDI clinical assessment tool. BDI depression scores significantly reduced over time, and at 1 year post-ceremony, 71% of the participants were in remission (BDI < 13) while 17% showed mild symptoms of depression (BDI 14–19). One year after the ceremony, 65% of the participants achieved a 50% reduction in depressive symptoms. After 1 month, the remission and response rates were somewhat lower (68% and 47%, respectively).

The present findings are in line with reports on ayahuasca’s benefits for clinically depressed patients. De Lima Osório et al. (2015) conducted an open-label trial in which they observed positive changes in HAM-D scores after ayahuasca intake in six clinically depressed patients. They found a reduction in depression ratings up to 82%, 21 days post-intake. Sanches et al. (2016) also found a clinical reduction in depression ratings in an open label trial with 17 patients with recurrent depression, lasting up to 21 days. In a first placebo-controlled randomized clinical trial, Palhano-Fontes and colleagues (2019) observed a stronger post-ayahuasca reduction in depression symptoms in 14 treatment-resistant depressed patients as compared to a placebo control group (*n* = 15), that lasted up to 7 days post-intake. All studies demonstrate the rapid and fast acting antidepressant effect of ayahuasca. The present study adds to these finding by suggesting that antidepressant effects of an ayahuasca analogue may last up to 1 year post-ceremony. Antidepressant effects appeared most prominent in individuals with mild to moderate symptoms of depression at baseline. Only one patient with a severe BDI score at baseline (BDI > 29) achieved remission against 11 patients with mild to moderate BDI ratings at baseline. A secondary analysis that included baseline BDI levels as covariate indeed confirmed that reductions in BDI varied between individuals as a function of baseline BDI, but interestingly also indicated that absolute decrements in BDI were largest in patients with high BDI scores at baseline. However, symptom reduction in patients with high BDI ratings at baseline was not always sufficient to exceed the 50% reduction level that defines a clinical response or to achieve remission. Overall, this seems to suggest that individuals with mild to moderate depression were more likely to achieve remission than individuals with severe symptoms of depression.

Response rates in the present study (55% day 1, 47% day 31, 65% day 365) were comparable to those reported by Palhano Fontes and colleagues (50% day 1, 64% day 7) in the ayahuasca group of their randomized clinical trial (RCT), even though current response rates were recorded at 1-month and 1-year follow-up. It is of interest that the ayahuasca analogue doses provided in the present study were relatively high. For example, DMT doses in previous placebo-controlled studies were 0.36 mg/kg (Palhano-Fontes et al. 2019), 0.75 mg/kg (Dos Santos et al. 2012; Valle et al. 2016), and 1 mg/kg (Dos Santos et al. 2011, 2012). In the present study, doses were not adjusted for body weight, but the average DMT dose for a 70-kg individual was 1.25 mg/kg. The latter was divided over 2 dose administrations, whereas in the former, studies comprised a single administration only. Mean subjective ratings of altered states of consciousness in the present study were moderate (between 16 and 54%) and comparable to other studies (e.g., Dos Santos et al. 2011, 2012; Uthaug et al. 2018; Palhano-Fontes et al. 2019; Valle et al. 2016), indicating that the current dosing regimen provided regular levels of altered states of consciousness.

On the secondary measures, DASS-21 scales showed a significant decrease in depression and stress ratings lasting up to 1 year post-ceremony. DASS-21 anxiety ratings reduced 1 month post-ceremony. Also, BSI-18 depression scores reduced significantly up to 1 year post-ceremony, and BSI-18 anxiety ratings reduced 1-day and 1 year post-ceremony. No change was found in self-reported somatization ratings. Also, 1 month and 1 year post-ceremony, participants reported significant increments in satisfaction with life. Furthermore, the FFMQ-15 mindfulness scales showed a self-reported increase in non-judging and non-reacting lasting up to 1 year post-ceremony. Mindful observing showed a similar pattern with an increase in the 1-day and 1-year follow-up, and mindful describing increased 1 year post-ceremony. The observed reductions in anxiety and stress lasting up to 1 year post-ceremony may be a logical consequence of the consistent reduction in self-reported depression and are in line with earlier findings (e.g., Bouso et al. 2012; Da Silveira et al. 2005; De Lima Osório et al. 2015; Van Oorsouw et al. 2021). Also, the increases in mindfulness, with increases in non-judging and non-reacting lasting up to 1 year post-ceremony, are in line with earlier findings (Murply-Beiner and Soar 2020; Soler et al. 2016; Soler et al., 2018) and are indicative for changes in one’s inner-judgments (e.g., self-criticism) and acceptance. The follow-up interview results are supporting this increase in self-acceptance and vulnerability.

Reductions in BDI depression scores correlated negatively with EDI and OB. This suggests that the psychedelic experience and more specifically the level of experienced ego dissolution and oceanic boundlessness are predictive of the positive mental health outcomes. Higher scores on both EDI and OB, two measures that refer to feelings of unity, spiritual insight, and bliss, correlated to lower depression scores post-ceremony, in line with earlier reports in healthy samples (Uthaug et al. 2018; Van Oorsouw et al. 2021). Ruffell et al. (2021) found a similar negative correlation between BDI scores and mystical experiences. It has been suggested that that acute reductions in hippocampal glutamate may play an important role in the experience of positive ego dissolution, via inducing a temporary loss of access to semantic autobiographical information, resulting in a subsequent breakdown of one’s personal identity (see Mason et al. 2020). Several participants in the present study reported 1 day post-ceremony, that they realized how stuck they were in rigid, destructive thought patterns: “I ran into my mental blocks,” “I was no longer isolated and locked up in my own mind,” “I got out of my defense and survival mechanisms,” “I saw the web of how everything is connected, and the returning patterns in my life, and I felt peace.” However, 1 year post-ceremony, EDI and OB no longer correlated with BDI scores. This suggests that other factors than the ayahuasca experience per se may have contributed to the sustained relief of depressive symptoms, such as enduring personal changes in lifestyle and attitude after integration or the involvement in additional mental health treatments. This is in line with results from a qualitative study by Callon et al. (2021), in which ceremony facilitators report that ceremony attendants who engage in complementary modalities such as psychotherapy, spiritual on contemplative practices, meditation, and “working” with insights have more successful ceremony processes and outcomes.

Being able to surrender to the psychedelic experience seems to be an important predictor for having a positive psychedelic experience, whereas mental preoccupation affects more challenging outcomes (Haijen et al. 2018; Russ et al. 2019). Lancelotta and Davis (2020) have shown that benefit enhancing strategies such as meditation and focus on intention were positively associated with the intensity of mystical type experiences, reduced the reported intensity of challenging effects, and increased ratings of spiritual significance, self-reported well-being, and life satisfaction (Lancelotta and Davis 2020). Watts and Luoma (2020) also emphasized the importance of preparing participants to enter the session with as little resistance as possible. In the present study, most individuals went through a trajectory of stopping their medication use and used ayahuasca analogue to help them recover from their depression. Many of them had been in years of therapy. Although baseline stress and anxiety levels were higher than in non-clinical samples (Uthaug et al. 2018; Van Oorsouw et al. 2021), overall, participants scored higher on the positive (OB) than on the challenging (AED) dimensions of 5D-ASC (see Fig. 5) and at comparable levels as reported in a non-clinical sample (Van Oorsouw et al. 2021). Participants in the follow-up interview all expressed trust in the facilitator of their ceremony and were appreciative of care and support that was provided, which may have created a positive setting in which positive ayahuasca experiences are more likely to occur.

A serious limitation of the present study is that there was no (treatment as usual) control group. Future studies should consider adding a control group. Not having a control group makes it difficult to isolate the pharmacological effect from expectancy and setting effects. In the present study, the majority of participants reported positive expectations in regard to the impact of the ayahuasca ceremony on their mental health status. A recent publication by Uthaug et al. (2021) has demonstrated the power of expectations and setting, especially in experienced ayahuasca users who showed mental health improvements while taking a placebo in a double blind study. Also, Palhano-Fontes and colleagues (2019) who conducted a randomized clinical trial to assess the antidepressant effect of ayahuasca in depressed patients reported response rate of 46% and 26% after 1 and 7 days in the placebo group. It therefore cannot be excluded that response rate in the present study may also have been affected by expectancies, a well-known psychological effect that is commonly reported anti-depressant studies (Kirsch et al. 2002), especially in patients with mild to moderate depression (Sparks et al. 2008). The inclusion of a control condition could provide more insight on not only how expectations may have affected the improvement in mental health but also what the effect of the ritual and setting is in absence of a pharmacological agent. Also, in the present study, an ayahuasca analogue was used, which is different from traditional ayahuasca concoctions (e.g., Palhano Fontes et al. 2019). However, also traditional ayahuasca brews show large variations in DMT and harmaline ratios (see Kaasik et al. 2021; Uthaug et al. 2018). Variations in constellations of ayahuasca or ayahuasca analogues however cannot be avoided as long as there is no agreement on a “standard recipe” for these brews. Another limitation of the current study was the lack of control over additional mental health approaches to which some of the participants were exposed. A total of nine participants took an additional psychedelic and three participants reported the use of antidepressant medication. Although additional psychedelic use did not correlate with BDI scores at 1 year post-ceremony, five participants reported subjective mental health benefits following additional psychedelic use. Additional confounding factors that may have contributed to the mental health ratings at the 1-year follow-up were the use of antidepressants, involvement in (psycho)therapy, and the initiation of self-directed mental health support routines. Therefore, it cannot be excluded that changes in BDI scores at 1-year follow-up are attributable to the ayahuasca experience alone. This notion seems also to be supported by a lack of correlation between BDI ratings and ayahuasca experience ratings at 1 year after the ceremony.

To sum up, the present study suggests that ayahuasca can relieve depression in clinically depressed patients, reduce anxiety and stress, and increase mindfulness capacities and quality of life up to one year after participation. For some, a single experience was sufficient, whereas others reported to have benefited from a second ceremony with ayahuasca or a different psychedelic. The intensity of the psychedelic experience correlated with the improvements up to 1 month post-ceremony. Almost half of the participants engaged in additional mental health treatments or programs during the course of study participation, which may have contributed to their improved mental health ratings at 1-year follow-up. The current findings add to the existing literature on the therapeutic potential of ayahuasca or its analogues (De Lima Osório et al. 2015; Palhano-Fontes et al. 2019; Ruffell et al. 2021; Sanches et al. 2016) and are a first step to more controlled clinical trials investigating the long-term antidepressant effects of ayahuasca and its analogues.

## Supplementary Information

Below is the link to the electronic supplementary material.

Supplementary file1 (DOCX 24.1 KB)
