# Supplementary material for: Therapeutic effect of an ayahuasca analogue in clinically depressed patients: a longitudinal observational study
Source: Psychopharmacology (Berl). 2022 Jan 24;239(6):1839–52. doi: 10.1007/s00213-021-06046-9 (PMC8785027; doi:10.1007/s00213-021-06046-9)
Supplement: Supplementary file 1 — Supplementary file1 (DOCX 24.1 KB) [file 213_2021_6046_MOESM1_ESM.docx]

Supplementary Materials

*Depression, Anxiety, and Stress Scale*

The Depression, Anxiety, and Stress Scale (DASS-21) is a shorter version of the originally 42-item long self-report questionnaire (Henry and Crawford 2005, Dutch translation; Wardenaar et al. 2017). Sample items for each subscale include the following; *“I couldn’t seem to experience any positive feeling at all”, “I was aware of dryness of my mouth”, and “I found it hard to wind down”*, respectively. The purpose of this scale is to measure constructs of depression, anxiety, and stress. The participants responded by rating the concordance with each statement from 0 *“did not apply to me at all”* to 3 *“applied to me very much, or most of the time”*. The subscale scores for depression, anxiety and stress are calculated by summing the scores for the relevant items, multiplied by 2, given that the original DASS has 42 questions. Cronbach alpha is good and was .95 in the current sample.

*Brief Symptom Inventory 18*
The Brief Symptom Inventory 18 (BSI-18) is a self-report scale which contains subscales on depression, anxiety, and somatization, (Derogatis 2001, Dutch translation; De Beurs, 2004). Participants were asked to rate a list of issues people can experience and the extent to which they experienced this over the past week (e.g. *‘feelings of worthlessness’*, ‘*suddenly scared for no reason’* and *‘pains in heart or chest’* for depression, anxiety and somatization respectively) on a 5-point Likert scale ranging from 0 *“none at all”* to 4 *“extremely”*. The BSI-18 is a reliable instrument for the assessment of psychological distress in both clinical and general populations with strong internal consistency and Cronbach’s alpha’s (α) of each sub-scale were .92, .87 and .66, for depression, anxiety and somatization, respectively.

*Satisfaction with Life Scale*The Satisfaction with Life Scale (SWLS) is a 5-item self-report scale (Diener et al. 1985). Sample items include: “*In most ways my life is close to my ideal”* and “*So far I have gotten the important things I want in life* “. The purpose of the scale is to assess someone’s satisfaction with life. The items are answered on a Likert-scale ranging from 1 “*Strongly disagree*” to 7 “*Strongly agree*”. The total score is obtained by summarizing the ratings from each item and ranges between 5 to 35, with higher scores indicating a greater life satisfaction. The scale has good psychometric properties with a Cronbach’s alpha coefficient (Beuningen 2012) which was .88 in the current sample.

*Five Facets Mindfulness Questionnaire-15*
This measure is a short form of the 39-item FFMQ (Baer et al. 2006, Dutch translation; Veehof et al. 2011). The FFMQ-15 is a self-report questionnaire which measures five different factors; 1) Observe: noticing experience that are both internal and external such as thoughts and emotions (e.g. *‘When I take a shower or a bath, I stay alert to the sensations of water on my body’*); 2) Describe: describing internal experiences (e.g. *‘I’m good at finding words to describe my feelings’*); 3) Acting with awareness: focus on the present activity (e.g. *‘I don’t pay attention to what I’m doing because I’m daydreaming, worrying, or otherwise distracted’*); 4) Non-judging: not evaluating or judging the present experience (e.g. *‘I believe some of my thoughts are abnormal or bad and I shouldn’t think that way’*); 5) Non-reacting: allowing thoughts and feelings to come without acting or reacting upon them (e.g. *‘When I have distressing thoughts or images, I “step back” and am aware of the thought or image without getting taken over by it.’*. The purpose of this scale is to obtain an understanding of an individual’s mindfulness related capacities. The participants answered the FFMQ-15 by rating the concordance with each statement on a 5-point Likert-scale that ranges from 1 “*never true*” to 5 “*very often or always true*”. The total FFMQ-15 score is obtained by adding all the sub-scale scores. Internal consistencies across sub-scales were between .84 and .93.

*Ego Dissolution Inventory*

The Ego Dissolution Inventory (EDI) is an 8-item self-report scale that assesses the participant’s experience of ego dissolution (Nour et al. 2016). Sample items for the scale includes the following: “*I experienced a dissolution of my “self” or ego”* and “*I felt at one with the universe*”. The purpose of this scale is to acquire a better understanding of the experiences the participants had about ego dissolution during the ayahuasca ceremony. The participants answered the scale with endpoints of either 0 = “*No, not more than usually*” or 100 = “*Yes I experience this completely/entirely*”. The EDI is scored by calculating the mean of all the 8 items (range 0-100). The higher the total score, the stronger the experience of ego dissolution. In this study a non-validated Dutch version was used (Cronbach *α* = .88).

*5-Dimensional Altered States of Consciousness Rating Scale*

The 5D-ASC is a 94-item self-report scale that assesses the participants’ alterations from normal waking consciousness (Dittrich et al., 2010; Studerus et al., 2010). The participant is asked to make a vertical mark on a horizontal 100 millimeter Visual Analogue Scale (VAS) to rate to what extent the statements applied to their experience in retrospect from 0: “*No, not more than usually*” to 100: “*Yes, more than usually*”. The 5D-ASC contains 5 key dimensions consisting of 11 subscales that identifies mystical-type experiences. The dimension *oceanic boundlessness* (OB; 27-items) measures derealization and depersonalization associated with positive mood states and correspond to the subscales, *experience of unity, spiritual experience, blissful state* and *insightfulness.* The dimension *anxious ego dissolution* (AED; 21 items) refers to ego disintegration and loss of self-control associated with anxiety. This dimension corresponds to the subscales: *disembodiment, impaired control and cognition,* and *anxiety*. A third dimension, *visionary restructuralization* (VR; 18 items) describes the visual aspects of the experience and refers to the subscales *complex imagery, elementary imagery*, *audio-visual synaesthesia* and *changed meaning of percepts*. The fourth and fifth dimensions are *auditory alterations* (AA; 16 items) and *reduction of vigilance* (RV; 12 items). In this study, a non-validated Dutch translation was used (see also Uthaug et al. 2019; Van Oorsouw et al. 2021). Cronbach’s alpha across sub-scales was .97. Based on previous findings (Roseman et al., 2018; Uthaug et al., 2019) we focused on 5D-ASC dimensions Oceanic Boundlesness (OB) and Anxious Ego Dissolution (AED), which might serve as interesting predictors of mental health outcomes.

References

Beuningen J (2012) The satisfaction with life scale examining construct validity. Statistics Netherlands 201209:3–23

De Beurs E (2004) Brief symptom inventory. Handleiding. Pits Publishers, Leiden (Netherlands)

Veehof MM, Peter M, Taal E, Westerhof GJ, Bohlmeijer ET (2011) Psychometric properties of the Dutch Five Facet Mindfulness Questionnaire (FFMQ) in patients with fibromyalgia. Clin Rheumatol 30:1045–1054

Wardenaar KJ, Wanders RBK, Jeronimus BF et al (2017) The Psychometric properties of an Internet- administered version of the Depression Anxiety and Stress Scales (DASS) in a Sample of Dutch Adults. J Psychopathol Behav Assess 1–16
